# Efficient authentication system based on blockchain for E-government

**DOI:** 10.1371/journal.pone.0336997

**Published:** 2025-12-05

**Authors:** Liang Liu, Kazumasa Omote

**Affiliations:** University of Tsukuba, Tsukuba, Japan; National Sun Yat Sen University, TAIWAN

## Abstract

With the rapid development of smart cities, e-government has emerged as a key application of information and communication technology (ICT). However, current e-government systems face challenges such as data redundancy and privacy breaches during the authentication process. To address these challenges, this research innovatively proposes an authentication scheme based on electronic identity (eID) cards and blockchain smart contracts. Users generate digital signatures using eID cards, and the smart contract automatically verifies the validity of the signature and checks the certificate status. The blockchain only stores government public keys for authentication, without retaining any user privacy data, thereby eliminating the risk of privacy breaches at the source. Additionally, this solution can automatically complete authentication without manual intervention, reducing the likelihood of human errors. It also provides a unified smart contract interface that can be widely applied in various e-government scenarios. Performance and security analyses indicate that this scheme can effectively resist common attacks and minimize privacy leakage risks, significantly enhancing the practical security of e-government systems.

## Introduction

With the development of smart cities, ICT has been extensively applied to enhance urban service efficiency and improve residents’ quality of life [1,2]. Among these applications, e-government stands out as a pivotal area of ICT utilization, facilitating the provision of various public services online through the Internet, including tax declaration, social security processing, and electronic voting [3–5]. However, prior to delivering these services, government departments must authenticate citizens’ identities through verification procedures. Currently, various government entities typically manage user identity data independently, leading to the repeated submission of personal information for identity registration and verification [6]. This decentralized management model not only increases data redundancy but also heightens the risks of data leakage. Cyberattacks and human errors further exacerbate personal data security vulnerabilities. Statistics indicate that there were 1,283 data breaches within the US government from early 2014 to 2023, resulting in nearly $30.4 billion in losses [7]. As e-government applications continue to expand, the demand for robust privacy protection in identity verification schemes has become increasingly urgent.

To prevent privacy breaches, traditional e-government authentication typically employs digital certificates and digital signatures to mitigate the risks associated with storing sensitive personal data [8,9]. On one hand, digital certificates generally record only issuance and usage-related information for identity verification, while certificate status information is stored in a centralized database. However, centralized management creates a single point of failure: if the database is compromised or malfunctions, certificate status information could become invalid [10]. Additionally, when a certificate is revoked or updated, synchronizing status information between departments in a timely manner can be challenging, which may lead to government agencies mistakenly recognizing revoked or invalid certificates, posing a significant threat of identity impersonation for users. On the other hand, electronic identity cards are often used to securely generate digital signatures, and these cards do not store information about users. However, digital signatures are susceptible to replication or replay attacks [11]. To enhance authentication security, some governments require in-person identity verification at designated locations [12]. Although this approach improves security, the reliance on fixed locations and manual intervention has resulted in a poor user experience and reduced efficiency. Consequently, designing an authentication scheme that simultaneously addresses security, efficiency, and user convenience has emerged as a critical challenge in the ongoing development of e-government.

To address these challenges, recent years have seen the field of e-government begin to explore the integration of blockchain technology [13]. Blockchain’s decentralized architecture helps mitigate single points of failure and data synchronization challenges in traditional systems. Furthermore, the distributed ledger structure of blockchain offers a tamper-proof data recording mechanism, thereby enhancing the integrity and credibility of the data. Additionally, the implementation of smart contracts allows for automated identity verification without human intervention, which significantly reduces the risk of human operational errors [14]. The integration of a challenge-response mechanism can also effectively prevent signature replay attacks, further safeguarding the security of the authentication process [15]. Moreover, smart contracts on blockchain platforms can establish unified interface standards and data formats for various government departments, promoting the universality and scalability of the solution and facilitating the deployment of applications across different scenarios [16]. However, some existing blockchain authentication schemes still necessitate the storage of sensitive information on the blockchain or in other databases, which poses a risk of privacy leakage.

This research proposes a novel e-government authentication scheme that innovatively integrates government-issued eID cards with blockchain smart contract technology. This solution achieves complete isolation of sensitive data during the authentication process, eliminating the need to store any personal privacy information and fundamentally reducing the risks of data leakage and privacy breaches.

The main contributions of this research are as follows.

- We propose an authentication system that combines eID cards with blockchain technology. The system prevents privacy leakage, minimizes on-chain storage requirements, and ensures efficient and secure authentication.
- We design and implement an automated authentication process that is compatible with multiple e-government agencies. Additionally, we conduct a security analysis against common attack vectors to demonstrate the robustness of the system.
- We evaluate the performance of the smart contract-based authentication mechanism, employing batch processing to optimize transaction execution time and identify the optimal batch size. The results indicate that our system performs efficiently in large-scale scenarios, demonstrating its feasibility for real-world deployment.

The structure of this paper is as follows: First, we delve into the intricacies of smart contracts and eID cards. Then, we review related works and clarify the progressiveness of this work. Next, we detail the proposed system architecture. Then, we evaluate the performance. At last, we discuss the advantages and security of the system.

## Preliminary

### Smart contract

The concept of smart contracts was first introduced by Nick Szabo in 1994 with the aim of facilitating digital markets [17]. A smart contract is a computer program that automatically executes contract terms. It is designed to carry out, control, or document legally relevant events or actions based on the stipulations of a contract or agreement. This automation reduces the need for intermediaries, streamlines processes, and minimizes the risks of errors or fraud. With the advancement of blockchain technology, smart contracts have been deployed on blockchains or Ethereum virtual machine. The inherent characteristics of blockchains, such as decentralization, immutability, and transparency, ensure the security and tamper-resistance of these smart contracts. Once a smart contract is deployed on a blockchain, it becomes immutable, meaning it cannot be altered or deleted. This ensures the integrity of the contract and guarantees that all parties involved can trust the execution of the contract. Furthermore, the decentralized nature of blockchains ensures that smart contracts operate in a trustless environment. Parties can engage in transactions and agreements without the need for central authorities or third-party verifications.

### eID card

The eID card, issued by governments or authoritative agencies, serves as a tangible proof of personal identity while also offering digital functionalities. In addition to identity recognition, eID cards are extensively used for online governmental procedures and more [18]. At the core of the eID card is its embedded microprocessor chip, which securely stores a wealth of digital data, including encryption keys, digital certificates, and personal details. This digital aspect of the eID card enables it to interact with electronic systems, making it a powerful tool for digital authentication and various electronic services. One crucial component stored in the eID card’s chip is the digital certificate, issued by a trusted certificate authority (CA), that includes the cardholder’s public key and other relevant details. Users also need to input a Personal Identification Number (PIN) to use the eID card, which ensures the secure identification and verification of the cardholder in digital environments. The adoption and functionalities of eID cards vary worldwide, with some countries fully integrating them into their societal framework for services ranging from online voting to e-government.

Many countries have used or plan to use the Elliptic Curve Digital Signature Algorithm (ECDSA) as signature algorithm [19,20].

## Related work

This research is closely related to two types of related work, authentication for e-government and authentication based on blockchain.

Ghosh *et al*. [12] proposed an e-government authentication scheme based on one-time password, citizenname and password, and biometric parameters. While this scheme provides strong security guarantees, it requires citizens to visit a designated location for authentication, which limits its practicality in remote scenarios. Soni *et al*. [21] integrated biometric authentication with elliptic curve cryptography to enable a flexible e-government authentication system on mobile devices. Although the proposed method effectively mitigates common attacks, mobile devices remain vulnerable to security breaches, which may compromise authentication integrity. Sharma *et al*. [22] introduced a lightweight smart card-based authentication protocol aimed at enhancing key security in authentication. However, the scheme lacks identity status management, allowing adversaries to exploit expired or revoked smart cards to bypass authentication.

Wu *et al*. [23] proposed a blockchain-based authentication system that stores verification data directly on the blockchain. While this approach ensures immutability and decentralization, it incurs high storage overhead and poses privacy leakage risks. Zyskind *et al*. [24] and Zhang *et al*. [25] designed hybrid on-chain and off-chain authentication systems that store certificate data in offline databases or the interplanetary file system while maintaining certificate index information on the blockchain. However, these systems rely heavily on the availability of external databases, raising concerns about system stability and data accessibility. Rahat *et al*. [26] developed an identity management system for government applications, where keys are stored in smart contracts, while identity data remains in local off-chain databases. However, the authentication process requires manual verification by personnel, making automated authentication infeasible. Mohanta *et al*. [27] introduced a smart contract-based authentication system that reduces the amount of citizen data stored on the blockchain, eliminating the need for offline databases. Authentication is performed via digital signature verification. However, this approach introduces identity impersonation risks, potentially undermining security.

The preliminary version of this paper was presented at IEEE International Conference on Blockchain 2023 [28]. This paper expands the application scope from the original focus on IoT device authentication to encompass e-government application scenarios, delving into the practical potential of blockchain and eID within the smart cities. Additionally, we have included a performance evaluation that analyzes throughput and authentication time across varying batch transaction sizes. Furthermore, a security analysis of the authentication process has been incorporated to enhance the robustness of our findings. Table 1 summarizes the key differences between the previous conference version and this extended journal version.

**Table 1:** Comparison between conference paper and this paper.

Feature	[28]	This Paper
Authentication Scenario	IoT	E-government
Challenge-Response Mechanism	✗	✓
System Interoperability	✗	✓
Threat Model	✗	✓
Testnet Performance Evaluation	✗	✓
Batch Transaction Optimization	✗	✓
Authentication Algorithms	✗	✓

The authentication systems of e-government and blockchain are mainly evaluated based on the following features. The feature comparison is summarized in Table 2.

**F1:** Securely stores citizen keys

**F2:** Supports remote authentication

**F3:** Immutable authentication records

**F4:** Real-time identity status management

**F5:** Verification without storing private data

**F6:** Automated authentication process

**F7:** Interoperability across organizations and agencies

**Table 2:** Feature comparison.

	F1	F2	F3	F4	F5	F6	F7
Ghosh *et al*. [12]	✗	✗	✗	✗	✗	✓	✓
Soni *et al*. [21]	✗	✓	✗	✗	✗	✓	✓
Sharma *et al*. [22]	✓	✓	✗	✗	✓	✗	✓
Wu *et al*. [23]	✗	✓	✓	✗	✗	✓	✓
Zyskind *et al*. [24]	✗	✓	✓	✗	✓	✓	✓
Zhang *et al*. [25]	✗	✓	✓	✗	✓	✓	✓
Rahat *et al*. [26]	✗	✓	✓	✗	✓	✗	✗
Mohanta *et al*. [27]	✗	✓	✓	✗	✓	✓	✓
Proposed system	✓	✓	✓	✓	✓	✓	✗

## Proposed architecture

### Overview

**Fig 1:**
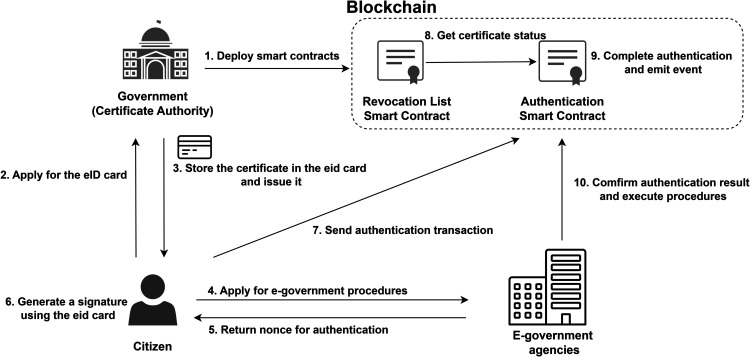
Overview of system architecture.

This system aims to achieve a secure and automated authentication process through a decentralized architecture that combines blockchain and eID cards, as shown in Fig 1. As a trusted CA, the government deploys a Revocation List(RL) contract and an authentication contract on the blockchain. The citizen obtains an eID card by going to the government and confirming their real identity. EID cards store citizen key pair, a certificate and have the ability to sign using the secret key. After applying for e-government procedures, the agency returns a random number as a challenge and monitors smart contract events. The citizen generates the signature of random number as a response, then submits the certificate and signature for authentication using a smart contract. Smart contracts automatically verify the validity of digital signatures and certificates, and trigger event notifications that record random numbers and results. The e-government agency determines whether the citizen’s authentication has been passed by confirming the content of the event, and thus decides whether to proceed with the procedures.

### Key points

#### Blockchain.

The government operates the blockchain as a consortium of permissioned nodes, representing multiple administrative levels such as national, prefectural, and municipal agencies. These nodes collectively maintain the ledger through a consensus protocol, which ensures that no single agency can unilaterally control or disrupt the system. Access to blockchain content and smart contracts is restricted to authorized citizens and organizations.

#### Challenge response mechanism.

A random number serves as a challenge, and citizens must sign it as a response. The authentication process records the citizen’s public key and the challenge to verify that the request originated from the same individual.

#### eID cards.

The eID card’s private key is securely protected and cannot be extracted, and its PIN cannot be tampered with. A valid PIN must be entered to activate the signature function, ensuring that signatures are generated only by the legitimate owner.

### Data flow

**Fig 2:**
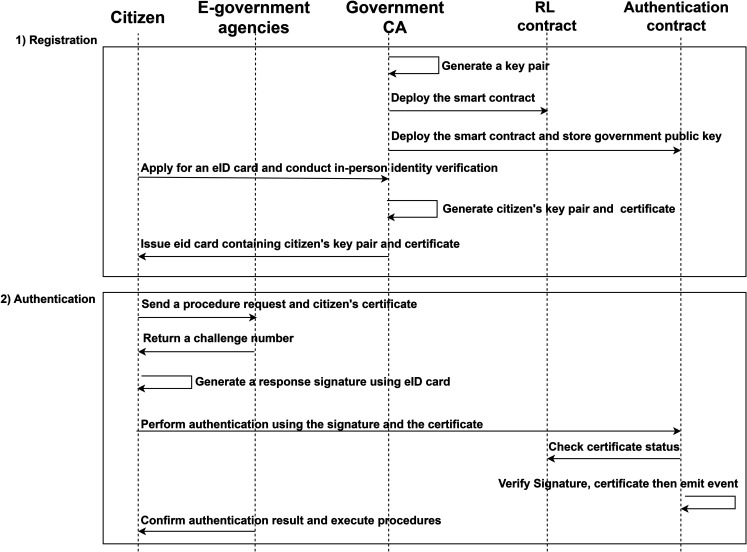
System data flow.

The proposed architecture consists of 2 steps, as shown in Fig 2. They are described using the following notations in Table 3.

**Table 3:** Notations description.

Notation	Description
*PK*_*ca*_	Government Public Key
*SK*_*ca*_	Government Secret Key
*PK*_*u*_	Public Key in User’s eID card
*SK*_*u*_	Secret Key in User’s eID card
*Cert*	User’s certificate
isRevoked	Certificate status
*N*_*c*_	Nonce of challenge
$sign_{u}(\cdot )$	Signing function by citizen’s secret Key
$\sigma_{r}$	Citizen’s signature for response
Verify(·)	Signature verifying

#### Registration.

The government first generates a key pair $PK_{ca},SK_{ca}$ for issuing certificates and then deploy two smart contracts to support authentication. The RL contract stores revoked certificates on the blockchain. If a certificate has been revoked, its status isRevokedissettoTrue; if no status exists, it defaults to *False*. The authentication contract stores the government’s public key, which can be viewed and modified only by the government. Similarly, only the government can update the revocation list periodically.


isRevoked∈{True,False}

Before using the system, citizens must visit a government office to apply for an eID card. Upon identity verification, the government begins creating the citizen’s eID card. It generates a key pair $\left(PK_{u},SK_{u}\right)$ and uses ECDSA to sign the citizen’s public key. Each citizen is assigned a unique key pair. The government generates certificates for citizens, including information about the certificate user, expiration date, and the government’s signature on the citizen’s public key *Cert*. The private key and certificate are then stored on the eID card, which is returned to the citizen.

If a citizen’s eID card is updated, lost, or stolen, the old version becomes invalid. The citizen can report this to the government, which then records the old certificate in the RL contract. The RL contract uses a hash table data format to store, update, and query revoked certificates, as shown in the Algorithm 1.

#### Authentication.

When citizens need to carry out e-government procedures, send a request to the relevant agency along with their certificate. The agency will check whether the citizen information in the certificate matches the identity of the procedure, and whether the certificate has expired. If the checks pass, the agency sends a random number *N*_*c*_ to the citizens as a challenge. The agency extracts the public key from the certificate, continuously monitors blockchain verification events, and waits for the citizen to complete authentication. Upon receiving the challenge, the citizen verifies their PIN and uses the eID card to generate a signature over the random number, as shown in the Algorithm 2.

The function Verify(·) for verifying signatures in the smart contract is shown in the Algorithm 3.

**Algorithm 1 Certificate storage algorithm.**

1: **Initialize** *certificate*

2: **Function** StoreRevokedCertificate(*certificate*)

3:   hash←keccak256(certificate)

4:   revokedCertificate[hash]←True

5: **End Function**

6:

7: //Query certificate status

8: **Function** GetStatus(certificate)

9:   hash←keccak256(certificate)

10:   **IF** hashexistsinrevokedCertificate **then**

11:    **return** revokedCertificate[hash]

12:   **ELSE**

13:    **return** False

14:   **END IF**

15: **End Function**

**Algorithm 2 Signature generation.**

**Require:** eID card, PIN, *N*_*c*_

**Ensure:** Digital signature

1: **Step 1: Unlock eID Card**

2: Citizen enters PIN

3: **if** PIN is correct **then**

4:   eID card is unlocked

5: **else**

6:   Access denied

7:   **Terminate Algorithm**

8: **end if**

9: **Step 2: Generate Digital Signature**

10: $\sigma \leftarrow sign_{u}(N_{c})$

11: **Step 3: Return Signature**

12: Output *σ*

**Algorithm 3 ECDSA signature verification.**

**Require:** Public key *PK*_*u*_, Signature *σ*, Content *c*

**Ensure:** Verification result (*True*/*False*)

1: **Step 1: Encode public key**

2: M←abiEncode(c)

3: **Step 2: Compute Keccak-256 hash**

4: $H_{M}\leftarrow \text{Keccak256}(M)$

5: **Step 3: Convert to Ethereum signed message hash**

6: $H_{\text{eth}}\leftarrow \text{toEthSignedMessageHash}(H_{M})$

7: **Step 4: Recover signer address**

8: $\text{signer}\leftarrow \text{ECDSA.recover}(H_{\text{eth}},\sigma )$

9: **Step 5: Compute address from public key**

10: $\text{computed\_address}\leftarrow \text{Keccak256}(PK_{u})[12:]$

11: **Step 6: Compare addresses**

12: **if** signer=computed_address **then**

13:   **Return** True

14: **else**

15:   **Return** False

16: **end if**

The algorithm begins by encoding the provided public key using ABI encoding. A Keccak-256 hash is then computed from the encoded data to generate a message hash. This hash is converted into an Ethereum-specific signed message hash format to ensure compatibility with ECDSA. The signature is subsequently used to recover the signer’s address using the ECDSA recovery function. In parallel, the Ethereum address corresponding to the provided public key is derived by applying the Keccak-256 hash function and extracting the last 20 bytes. Finally, the recovered signer address is compared with the computed address; if they match, the signature is verified as authentic, otherwise, the verification fails.

Citizens verify their identity by providing the citizen public key, random number, certificate, and response signature to the smart contract. The contract first checks the certificate status via the RL contract. If not revoked, it verifies the certificate using the CA’s public key, and then validates the citizen’s signature over the challenge. Upon successful verification, the contract emits an event recording the citizen’s public key and the challenge number. As shown in the Algorithm 4. This event is stored on transaction log and can be accessed by members in the blockchain.

**Algorithm 4 Citizen authentication in smart contract.**

**Require:** *PK*_*u*_, *N*_*c*_, *Cert*, $\sigma_{r}$

**Ensure:** transaction event

1: **Step 1: Retrieve Certificate Status**

2: isRevoked←GetStatus(Cert)

3: **Step 2: Check Revocation Status**

4: **if** isRevoked=True **then**

5:   **Return** "Authentication Failed"

6: **end if**

7: **Step 3: Verify Certificate**

8: **if** $Verify(Cert,PK_{ca},PK_{u})=\text{False}$ **then**

9:   **Return** "Authentication Failed"

10: **end if**

11: **Step 4: Verify User Signature**

12: **if** $Verify(\sigma_{r},PK_{u},N_{c})=\text{False}$ **then**

13:   **Return** "Authentication Failed"

14: **end if**

15: **Step 5: Authentication Successful - Trigger Event**

16: EmitEvent($PK_{u},Success,N_{c}$)

The agency immediately compares the event record with the original request. Once the public key and random number match, it confirms successful authentication and proceeds with the e-government procedures.

## Performance evaluation

We utilize Remix for developing smart contracts, with the contract code written in Solidity. The local blockchain network is deployed using the Hardhat framework, operating a single-node blockchain. The testing environment consists of a machine equipped with an Intel Core i5-9600KF CPU and 32GB of RAM. To evaluate smart contract performance under different transaction loads, we used JavaScript to generate multiple blockchain accounts that simultaneously sent authentication requests. Additionally, the contracts were tested on the Ethereum Sepolia test network, with deployments and transactions executed via MetaMask.

### Gas consumption

Gas consumption is a critical factor in evaluating the efficiency and feasibility of blockchain-based authentication systems. Gas primarily measures the computational resources required for executing transactions or smart contracts. Table 4 presents a comparative analysis of the gas costs associated with key operations of our proposed system under different environments on the blockchain.

**Table 4:** Comparison of gas consumption.

	Hardhat	Sepolia	[29]
Revocation List Contract Deployment	238,636	217,594	/
Revoked Certificate Storage	44,690	45,738	/
Authentication Contract Deployment	938,397	1,066,810	1,560,740
Public Key Storage	44,112	44,054	/
Authentication	53,274	50,803	97,213

The gas consumption on the local blockchain is nearly identical to that observed on the test network. Compared to Nyame *et al*.’s system, our system reduces deployment costs by approximately 31.6%, demonstrating lower on-chain overhead. Furthermore, authentication costs are reduced by 47.7%, highlighting the efficiency of our smart contract logic in verifying citizen identity.

### RL contract performance

In this subsection, we evaluated the performance of storing certificates in the RL contract. Given the high-frequency transactions in e-government applications, we also examined the performance of batch transactions. Batch transactions refer to processing multiple sub-transactions within a single transaction request, rather than submitting each transaction individually.

We evaluated performance under transaction counts of 1, 10, 100, 1,000, 5,000, and 10,000, with batch sizes of 1, 10, 50, 100, and 600, respectively. On the Ethereum blockchain, the gas limit per block is 30 million, while the gas consumption for a single storage operation is approximately 45,000. A batch size of 600 approximates the theoretical maximum number of transactions a single Ethereum block can process, offering a practical upper bound for real-world deployment considerations.

**Fig 3:**
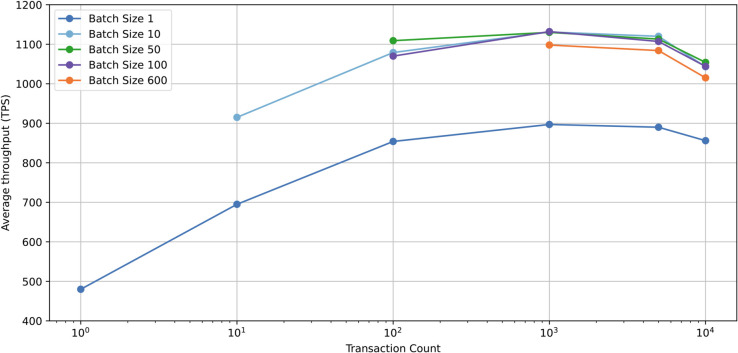
Average throughput of certificate storage in different transaction count.

Fig 3 illustrates the relationship between transaction throughput (transactions per second, TPS) and the number of transactions processed under different batch sizes in the proposed blockchain-based authentication system. Batch processing significantly improves transaction throughput compared to individual process. Specifically, when batch size increases from 1 to 50, the TPS increases markedly, indicating substantial performance optimization benefits from batching transactions. However, further increasing the batch size results in diminishing marginal improvements, batch sizes exceeding 100 result in decreased throughput. Additionally, the stable TPS demonstrates its capability to handle extensive authentication workloads efficiently.

### Authentication performance

The authentication function is the core component of the system. To evaluate the impact of batch processing on the execution performance of the smart contract, we conducted tests on the contract’s performance under different transaction counts.

**Fig 4:**
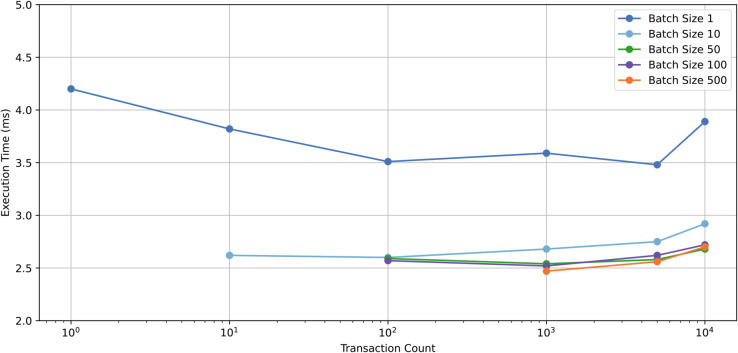
Average execution time of authentication in different transaction count.

The execution times for various transactions are illustrated in the Fig 4. Similarly, the gas consumption for a single authentication operation is approximately 50,000. A batch size of 500 simulates the maximum number of transactions that a single Ethereum block can handle.

**Fig 5:**
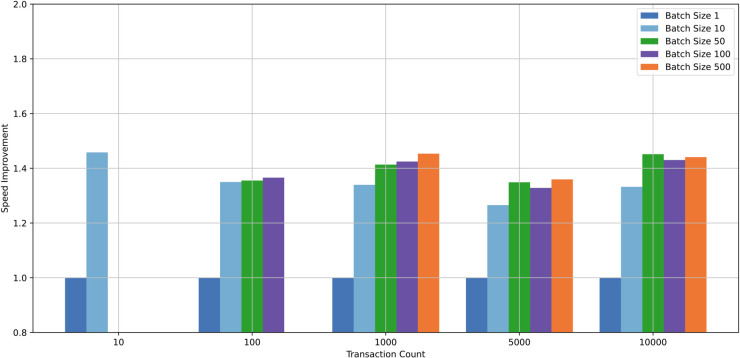
Authentication speedup of different batch size.

While the execution time for individual transactions decreases as the number of transactions increases, it remains above 3.5 ms. In contrast, batch processing significantly reduces execution time, with all batch-processed transactions completing in under 3 ms. The performance improvements achieved through batch processing, relative to individual transaction execution, are also visualized in the Fig 5.

Batch processing consistently provides a speedup of at least 1.2× but does not exceed 1.5×. The optimal batch sizes vary depending on the transaction count:

- For low transaction counts (10), small batch sizes (10) or individual transaction execution are more efficient.
- For medium transaction counts (100-500), a batch size of 100 yields the best performance.
- For high transaction counts (more than 1,000), larger batch sizes are preferable.
- At a transaction count of 1,000, a batch size of 500 achieves the best performance, with an execution time of 2.47 ms.

## Discussion

### Advantages

Citizens can securely perform authentication from any geographic location without the need to access designated facilities. They can generate signatures using their eID cards on mobile devices or by connecting a card reader to various devices. This approach significantly reduces the constraints associated with traditional methods that require designated authentication locations or specialized hardware, thereby enhancing accessibility and convenience across diverse real-world scenarios.

Our system demonstrates high scalability through its architectural design. By leveraging the modular nature of blockchain and a smart contract-driven approach, it allows for the integration of additional authentication protocols and various identity types. This adaptability is crucial for meeting the evolving demands of e-government applications. The system’s structure further reduces storage and computational overhead per transaction, supporting parallel execution in high-concurrency environments. In addition, the design separates policy logic from protocol logic, allowing adaptation to different regulation.

Universality is a key advantage of our system. It employs standardized protocols and frameworks to ensure interoperability among different organizations and government agencies. This guarantees widespread adoption of the authentication system, facilitating seamless collaboration across various e-government platforms and service providers.

Our architecture separates governance from authentication. While central government agencies define certificate issuance policies, the verification process is fully decentralized. Authentication is performed through immutable smart contracts deployed on the blockchain, enabling any authorized party to independently verify certificates without relying on a central server or higher-level administrative approval. This design enhances the system’s flexibility and scalability in its authentication infrastructure.

### Security

In this subsection, a security discussion is conducted on common attacks.

#### Threat model.

We assume that the government operates as a fully trusted entity for key management and authentication. However, the system is designed to maintain integrity and availability through a consensus-based architecture, even in the event of compromise or unavailability of some governmental nodes.

During eID card issuance, the government ensures consistency among the certificate, the eID card, and the citizen’s real-world identity. Additionally, the government functions as blockchain nodes, managing the execution of smart contracts and blockchain data in accordance with the consensus protocol. It also has access to the input and output information of smart contracts.

Users in the system include both honest participants and potential malicious actors. Malicious citizens may attempt to exploit vulnerabilities by obtaining signatures from other citizens or conducting identity spoofing attacks based on publicly available information.

Attackers can be external adversaries or internal malicious citizens seeking to impersonate legitimate citizens to pass authentication. However, their computational capabilities are limited, preventing them from deriving private keys from corresponding public keys.

#### Replay attack.

The RL smart contract records the real-time status of certificates, ensuring the validity of government-issued identity authorizations. Each signature is uniquely associated with a specific request and cannot be reused.

The system can detect signatures generated by invalid eID cards based on the certificate status. Furthermore, even if an attacker submits an outdated signature that previously passed authentication, agencies can still verify the requester’s identity by comparing the consistency of the associated random number.

#### Impersonation attack.

Each procedure request is assigned a unique random number. Citizens generate a signature for this random number using their eID cards. The eID card securely stores the citizen’s cryptographic keys, preventing attackers from extracting the private key. Since generating a valid signature requires both the citizen’s eID card and their PIN code, it is impossible for an attacker to forge a signature and impersonate another individual.

An attacker might attempt to authenticate using their own certificate and eID card. However, the random number associated with the transaction request is sent exclusively to the intended citizen. Therefore, attackers are unable to obtain valid response signatures generated by random numbers.

Attackers are unable to generate effective responses by exhaustively enumerating all possible values, Every authentication is recorded on the blockchain, and excessive authentication requests will be discovered by nodes.

Moreover, attackers cannot launch any blockchain-based attacks to manipulate the data stored in the smart contract or modify the result of authentication. Since the blockchain nodes are operated by a trusted government entity, attackers are unable to control more than 51% of the computational power, effectively preventing majority-based attacks.

### Limitation and future work

While our system achieves decentralized verification through smart contracts and permissioned blockchain nodes, certain aspects of identity management remain centralized. The current trust model places the government at the core of key generation, certificate issuance, and revocation processes. In the context of e-government, where identity authentication involves sensitive citizen data, centralized oversight is often necessary to ensure regulatory compliance, procedural standardization, and legal accountability.

To guarantee data integrity and availability, the system adopts a consortium blockchain architecture in which government nodes participate in the consensus process. This setup improves fault tolerance but does not eliminate the risks associated with potential miscoordination or misuse of authority by the central entity.

Future research may explore inter-governmental consensus protocols and federated governance models to further decentralize administrative control while preserving legal safeguards. Additionally, techniques such as threshold-based certificate issuance, multi-party certificate authority governance, and self-sovereign identity frameworks such as featuring decentralized identifiers and verifiable credentials can be leveraged to distribute trust and enhance user autonomy in digital identity systems.

Related work [13,30] has demonstrated the feasibility of using the Japanese eID card to generate digital signatures and verify them via Ethereum smart contracts, supporting both RSA and ECDSA algorithms. These findings indicate that technical integration with real-world eID cards is viable, particularly in systems like ours that avoid on-chain user data storage and rely on signature verification. Nonetheless, cross-jurisdictional regulatory and procedural differences necessitate significant adaptation for deployment in specific national contexts.

We also recognize that our current performance evaluation focuses primarily on gas consumption and smart contract execution time. Although related studies have measured client-side authentication latency (typically between 0.3 and 0.8 seconds), we have not yet conducted evaluations of scalability or fault tolerance [30].

The current evaluation was conducted in a single-node environment. Key real-world performance metrics require multi-node deployment and distributed testing, such as end-to-end user authentication latency, system throughput under concurrent requests, and fault tolerance in the event of node or contract failure. These metrics are also heavily influenced by factors such as network topology, inter-node latency, bandwidth constraints, and the choice of consensus protocols. We identify multi-node evaluation and cross-platform deployment as important directions for future research.

## Conclusion

This article proposes an authentication scheme based on blockchain smart contracts and eID cards to address the significant risks of data leakage, insufficient privacy protection, low efficiency in manual verification, and challenges in cross-departmental authentication prevalent in current e-government authentication schemes. This scheme achieves privacy protection by utilizing blockchain technology to store only government public key information, thereby avoiding the retention of any sensitive user privacy data. Concurrently, smart contracts facilitate the automatic real-time synchronization of certificate status and signature verification without manual intervention, effectively mitigating security risks associated with human operations and enhancing the efficiency of authentication. Furthermore, this scheme has established a unified standard for smart contract interfaces, enabling broad and convenient application in authentication scenarios across different government departments. Performance evaluation results indicate that this scheme significantly reduces transaction execution time and gas costs, while efficiently supporting large-scale deployment. Additionally, security analysis demonstrates that this scheme can effectively counter typical threats such as replay attacks and simulation attacks, thereby enhancing the security and reliability of authentication.

## Supporting information

S1 Table Raw data underlying Fig 3. (XLSX)

S2 Table Raw data underlying Fig 4. (XLSX)

S3 Table Raw data underlying Fig 5. (XLSX)
