# Correction: Efficient authentication system based on blockchain for E-government

**DOI:** 10.1371/journal.pone.0344391

**Published:** 2026-03-05

**Authors:** Liang Liu, Kazumasa Omote

The Funding statement for this article is incorrect. The correct Funding statement is as follows: This work was funded by JSPS KAKENHI, Grant Numbers 23K24844 and 25H01106, awarded to KO. The funders had no role in study design, data collection and analysis, decision to publish, or preparation of the manuscript.
